# Sources of Microbial and Organic Contaminants in the Production of Soybean Whey Protein for Feed and Potential Food Applications

**DOI:** 10.1002/fsn3.71709

**Published:** 2026-04-29

**Authors:** Yuanxiang Liu, Wei Wang, Yishu Peng, Luhua Feng, Chongzhou Li, Zhiyong Zhang, Junhui Zhao, Chunwen Yang, Tong Mu, Jinlu Wang, Chunfang Li, Chunyu Yang

**Affiliations:** ^1^ State Key Laboratory of Microbial Technology, Institute of Microbial Technology Shandong University Qingdao Shandong China; ^2^ Shandong LvBang Bio‐Tech Company Binzhou China

**Keywords:** anaerobic rancidity, *Enterococcus faecium*, *Megasphera*, soybean whey protein (SWP), soybean whey wastewater (SWW), sustainable production

## Abstract

Soybean whey wastewater (SWW), a rich source of soybean whey protein (SWP), is prone to microbial rancidity, posing environmental and resource challenges. This study explores the causes of rancidity—characterized by a pungent, sour, and putrid odor—in the effluents of sealed buffer tank during SWP recovery via pneumatic flotation. Metagenome, bacterial diversity, and HPLC analyses showed the obligate anaerobe *Megasphaera* spp. dominated rancid effluents (up to 44% abundance), consumed lactate (decreasing from 10.2 g/L in influent to 2.7 g/L in effluent), and produced malodorous propionate and butyrate (up to 3.6 and 4.3 g/L, respectively). Three mitigation strategies were assessed: (1) full‐scale high‐throughput aeration—likely effective but energy‐ and cost‐intensive; (2) local aeration—low‐cost but weakly inhibitory; and (3) microbial intervention using the probiotic *Enterococcus* *faecium* LBSW, which colonizes the buffer tank, with localized aeration used only if microbial control fails. Strategy (3) was adopted for its energy and cost efficiency, successfully reducing pollution and supporting SWP recovery. Although the biosafety of *E. faecium* LBSW in food applications requires caution, the recovered SWP is primarily intended for animal feed, and subsequent high‐temperature drying and sterilization (> 120°C) also offer potential for food‐grade use.

## Graphical abstract

Schematic illustration of an integrated strategy for soybean whey wastewater (SWW) treatment combining high‐throughput air flotation sorting with microbial monitoring and interaction analysis. This dual approach enables efficient nutrient recovery from polysaccharide‐ and protein‐rich SWW, eliminates rancid pollution, and achieves sustainable nutrient recycling with precise pollution source control at reduced cost. The recovered nutrients show promising applications in animal feed and potential food ingredients.

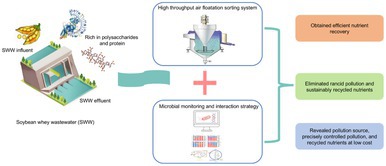

## 1 Introduction

With the substantial growth of the global population, the demand for nutrition, especially protein, is increasing (Aiking 2011; Kim et al. 2019). As one of the most important protein sources for livestock feed and human consumption, the industrial scale of soybean protein manufacture is increasing worldwide (Thrane et al. 2017; Messina 2022). Soybean whey wastewater (SWW), a byproduct of soybean protein isolate (SPI) production (Su et al. 2011; Wang and Serventi 2019), is a nutrient‐dense liquid—particularly rich in soybean whey protein (SWP) (Yu et al. 1998; Qiu et al. 2019). This protein fraction is highly valued in the feed and food industries due to its exceptional nutritional profile and diverse applications (Feng et al. 2009; Lee et al. 2014). Consequently, the effective utilization of SWW has become a focal point of feed and food industrial interest in recent years (Feng et al. 2009; Lee et al. 2014).

**FIGURE 1:**
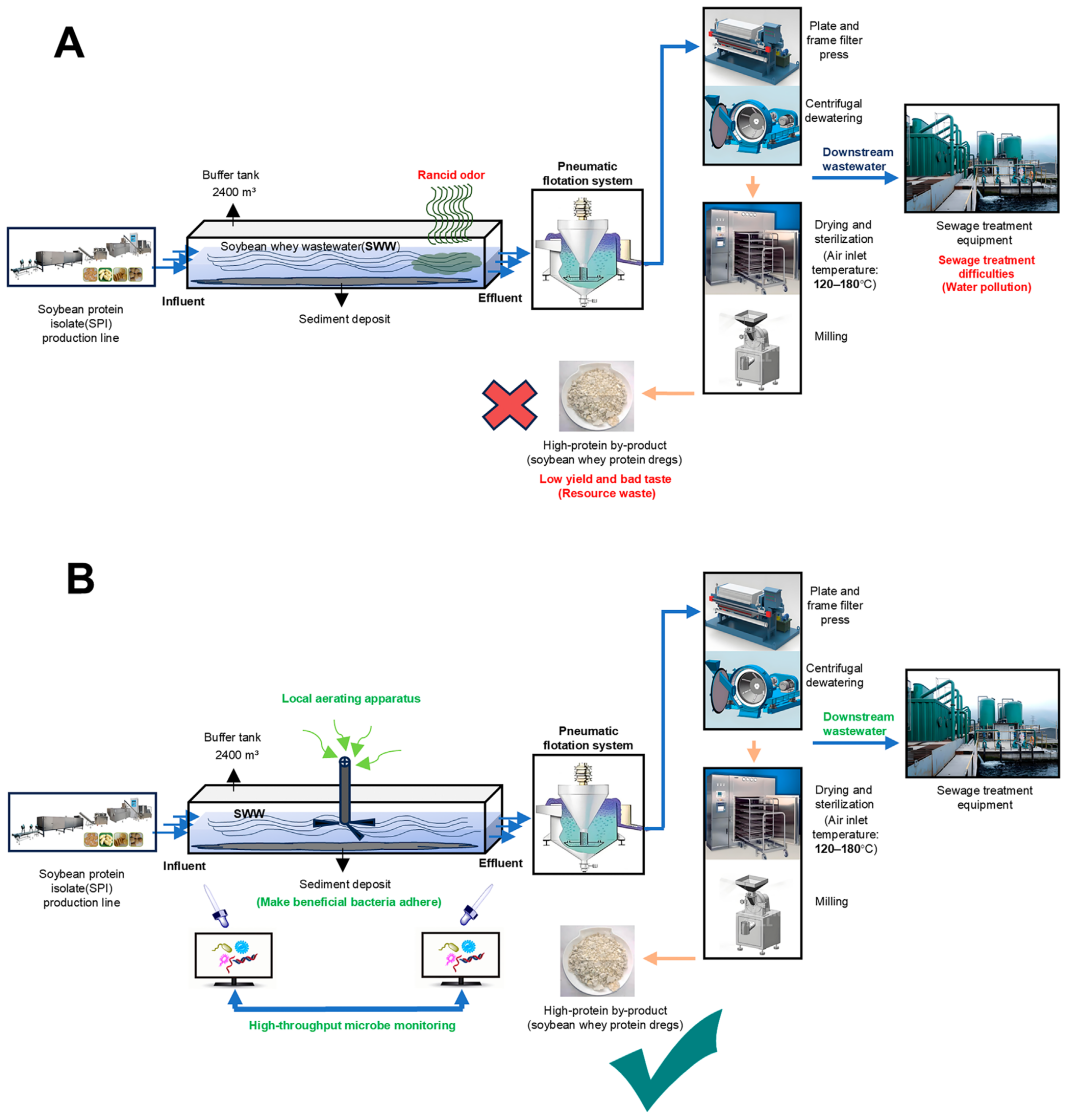
Schematic comparison of conventional (A) and improved (B) soybean whey wastewater (SWW) treatment processes. (A) Conventional treatment process: soybean whey wastewater (SWW) from the soybean protein isolate (SPI) production line enters a 2400 m^3^ buffer tank, where rancid odor develops due to microbial spoilage. The wastewater undergoes pneumatic flotation to remove floating solids, followed by dewatering via plate‐and‐frame filter press or centrifugal dewatering (stacked screw dewatering). The resulting high‐protein by‐product (soybean whey protein dregs) has low yield and poor taste, leading to resource waste. Smelly downstream wastewater is discharged to sewage treatment equipment, posing challenges for water pollution control. (B) Improved treatment process: to enhance oxygenation and prevent anaerobic spoilage, a local aeration apparatus is installed in the buffer tank alongside the addition of colonizing beneficial microorganisms, both working in tandem to reduce rancidity. This enhances microbial stability and reduces foul odor. High‐throughput microbe monitoring is implemented to track microbial community dynamics, enabling process optimization. The same downstream processing steps (filtration, dewatering, drying, sterilization, and milling) are applied, yielding a higher‐quality high‐protein by‐product with improved flavor and reduced waste. The overall system achieves better environmental performance and resource utilization.

Pneumatic flotation system is currently the predominant method for recovering SWP from wastewater (Li et al. 2018; Zhou et al. 2020). This technique is renowned for its high throughput and efficiency, making it an ideal choice for industrial‐scale operations. As shown in Figure 1A, at Shandong Lvbang Biological Co. Ltd. (dedicated to the resource utilization of SWW), the front end of the pneumatic flotation system is an open‐air sealed buffer tank (40 m × 20 m × 3 m, 2400 m^3^, with water temperature maintained at approximately 40°C and pH 3–5). Upstream of the buffer tank is the SWW generated from SPI process (influent). The bottom of the tank contains bioactive sludge, providing colonization sites for microorganisms. The downstream of the buffer tank is termed the effluent, directly connected to the pneumatic flotation system. After entering the pneumatic flotation system, the effluent undergoes plate‐and‐frame filtration or centrifugal dewatering (stacked screw dewatering) to separate the solid phase. The solid phase is then dried and sterilized (inlet air temperature: 120°C–180°C) and milled to form feed‐grade okara. This okara is rich in crude protein (25%–35% SWP), fiber, and prebiotics (e.g., residual oligosaccharides), making it a high‐quality raw material for animal feed with potential applications in the food industry. However, during the SWP recovery process, SWW effluents frequently suffer from microbial rancidity (up to four months in a year), producing a strong, pungent, foul odor, contaminating the SWP products with an offensive odor (Chua and Liu 2019) (Figure 1A). These issues not only hinder the production of SWP but also compromise the quality and safety of the end products, thereby posing significant challenges to the feed and food industries.

Therefore, elucidating the dynamic changes in microbial communities during the SWP production process and identifying potential solutions are of paramount importance. This study aims to investigate the causes of microbial spoilage (rancidity), including the specific microorganisms and substances responsible, and to explore cost‐effective, energy‐efficient, and viable solutions (Figure 1B). By achieving these objectives, we intend to optimize the yield and quality of SWP products, thereby contributing to the sustainable development of the feed and food industries.

## 2 Material and Methods

### 2.1 Materials, Strains, and Media

Influent and effluent samples of SWW were collected from the SWW buffer tank of Shandong Lvbang Bio‐tech company, Binzhou, P. R. China (37°14′83´´N 118°18′45″ E). Organic acids were all purchased from Aladdin Reagent Co. *Enterococcus* *faecium* LBSW was stored in our laboratory and deposited in the China General Microbiological Culture Collection Center, with the accession number of CGMCC No. 25,299 (Subsequent full‐length 16S rDNA sequencing confirmed that it was actually *Enterococcus faecium* SF68). MRS medium was used for the isolation and routine cultivation of *E. faecium* LBSW. YCFA medium was used for the isolation of *Megasphera* sp. LY from rancid SWW (Liu et al. 2007). MRS medium with 10 g L^−1^ of sodium lactate addition was used for its routine cultivation. All operations for the culturation of *Megasphera* sp. LY were under anaerobic conditions.

### 2.2 Sampling and Analytical Methods

Given the lack of prior knowledge regarding the specific microbial or chemical causes of rancidity in SWW effluent, samples were initially grouped based on observable and consistent sensory characteristics—specifically, the intensity of a pungent, sour, and putrid odor (rancidity)—as this represents the primary operational concern in industrial settings. A trained five‐member panel (all members have worked at Shandong Lvbang Bio‐tech company for over 3 years) evaluated all samples using a standardized four‐point scale (− = none, + = slight, ++ = moderate, +++ = strong), and the degree of rancidity in the samples was subsequently averaged, with every three samples exhibiting the same rancidity level grouped together (Table 1). This phenotypic stratification enabled a targeted comparative analysis to uncover the underlying microbial and metabolic mechanisms, which were subsequently identified through metagenomics and High Performance Liquid Chromatography (HPLC) analysis.

**TABLE 1:** Characteristics of SWW influent and effluent groups.

Group	Rancid odor level	pH	DO (mg L^−1^)	COD_cr_ (mg L^−1^)
UI	–	4.53 ± 0.15	6.12 ± 0.32	13902.5 ± 435.5
SI	–	4.76 ± 0.18	5.54 ± 0.26	12115.0 ± 510.4
MI	–	4.49 ± 0.11	5.73 ± 0.40	16800.0 ± 831.2
CI	–	4.58 ± 0.14	5.53 ± 0.37	17151.6 ± 484.7
UE	–	4.01 ± 0.12	3.70 ± 0.22	10816.3 ± 941.2
SE	+	4.14 ± 0.17	1.85 ± 0.14	8730.0 ± 368.2
ME	++	3.82 ± 0.15	1.19 ± 0.18	12940.0 ± 447.5
CE	+++	4.12 ± 0.13	0.76 ± 0.06	13488.6 ± 387.0

*Note:* − means no rancidity and + means rancidity. Sample grouping was solely dependent on the intensity of putrid odor; three samples with identical rancidity levels were grouped together. UI, SI, MI, and CI are the influent samples, which correspond to the effluent samples UE, SE, ME, and CE, respectively. UE represents effluent samples of SWW without rancidity, SE for effluent samples of slightly rancid SWW, ME for effluent samples of moderately rancid SWW, and CE for effluent samples of severe rancid SWW. All the influent samples have no rancid odor, but the effluent samples have different degrees of rancid odor.

The SWW influent and effluent samples were collected from the mid‐liquid point of the buffer tank inlets and outlets, respectively. Based on the sampling locations and the rancidity levels assessed by the personnel, all samples were categorized into eight groups (Table 1). UI, SI, MI, and CI are the SWW influent groups, which correspond to the effluent groups UE, SE, ME, and CE, respectively. UE represents the effluent group without rancidity, SE for the effluent group with slight rancidity, ME for the effluent group with moderate rancidity, and CE for the effluent group with severe rancidity. All the influent samples have no rancid odor, but the effluent samples have different degrees of rancid odor. The pH of SWW was measured by the pH meter (Mettler), dissolved oxygen (DO) was detected by the dissolved oxygen electrode (Mettler), and the Chemical Oxygen Demand (COD_cr_) was determined by the potassium dichromate method (Dedkov et al. 2000).

For organic acids detection, all samples were centrifuged at 13,400 × g for 10 min and the supernatant was filtered through a 0.22 μm filter. The concentration of organic acids was determined by a Shimadzu C18 column (VP ODS 250*4.6/5 μm) on the Shimadzu HPLC system (LC 20 AT) equipped with a UV detector. The mobile phase was 1% trifluoroacetic acid and 20% acetonitrile in water at a flow rate of 0.5 mL min^−1^, and the column temperature was 40°C (Zheng et al. 2024).

### 2.3 High‐Throughput 16S rDNA Sequencing of Influent and Effluent SWW

All samples for high‐throughput sequencing were centrifuged at 6000 × g, the resulting solids were frozen on dry ice before high‐throughput 16S rDNA sequencing (16S rRNA gene amplicon sequencing). The V3‐V4 region of bacterial 16S rRNA gene was amplified by polymerase chain reaction (PCR) with primers 338F (5‐ACTCCTACGGGAGGCAGAG‐3) and 806R (5‐GGACTACHVGGGTWTCTAAT‐3) (Zhang et al. 2016) in the ABI GeneAmp9700. Sequencing was performed by an Illumina Miseq PE 300 high‐throughput sequencer at Majorbio Corporation (Shanghai, China) and analyzed on the Majorbio I‐Sanger cloud platform (http://www.i‐sanger.com/). The data have been deposited in the National Center for Biotechnology Information (NCBI) database, with accession number BioProject PRJNA1012818.

### 2.4 Shotgun Metagenomic Sequencing

For the construction of paired‐end library, the DNA sequences were randomly fragmented to an average length of approximately 400 bp by Covaris M220 (Gene Company Limited, China). The paired‐end (PE) library was constructed using NEXTFLEX Rapid DNA‐Seq kit (Bio Scientific, Austin, TX, USA) and sequenced by an Illumina Hiseq 2000 platform (Majorbio Corporation, Shanghai, China), according to the 101 bp paired‐end sequencing strategy. Sequence reads of low quality or ambiguous were removed, and the pair‐end sequence reads were then merged into tags. The average length of tags was about 170 bp. The data have been deposited in NCBI, with accession number BioProject PRJNA1012830. The metagenomic sequencing results were analyzed using the Majorbio I‐Sanger cloud platform (http://www.i‐sanger.com/).

### 2.5 Strains Isolation

For *Megaphera* strain isolation, the severely rancid SWW effluent was serially diluted and spread on the YCFA agar medium supplemented with 10 g L^−1^ of sodium l‐lactate, and then placed in an anaerobic bag, sealed, and cultivated at 40°C for 7 d, after which colonies with black‐green color were picked up and purified. The genomic DNA of *Megaphera* was isolated by the genomic DNA isolation kit and used as the template for the amplification of 16S rRNA gene, using the primers of 27F and 1492R. The PCR product was purified, ligated with the pEASY‐Blunt vector, and transformed into the *E. coli* DH5α competent cells. Positive transformants were first screened by PCR and then sequenced by Sangon Biotech Co. Ltd., Shanghai, China.

### 2.6 Co‐Cultivation of *Enterococcus* *faecium* LBSW With *Megasphera* sp. LY

Both isolates *E*. *faecium* LBSW and *Megasphera* sp. LY were cultured in the MRS mediums to an optical density (OD_600nm_) of 2.0 under anaerobic conditions. The co‐cultivation system was 2% (v/v) of *Megaphera* sp. LY and 0%, 5%, 10%, 15%, or 20% (v/v) of *E*. *faecium* LBSW respectively, in a 500‐mL flask containing 100 mL of SWW influent. Each group performed in triplicates. After statically cultivating at 40°C for 3 d, the co‐culture odor of every group was smelled by a trained five‐member panel, and the severity of the rancid odor was recorded. In addition, treatments Ino‐0 (2% *Megasphera* sp. LY and 0% *E. faecium* LBSW) and Ino‐15 (2% *Megasphera* sp. LY and 15% *E. faecium* LBSW) were sampled for organic acid analysis and shotgun metagenomic sequencing.

## 3 Results and Discussion

### 3.1 Metagenomic Analysis Revealed Anaerobic Condition as the Driver of SWW Effluent Rancidity

Given that the specific microbial agents and metabolic products responsible for rancidity in SWW were unknown at the outset of this study, we employed rancid odor intensity as a practical, operationally relevant phenotypic proxy for rancidity severity. In industrial practice, off‐odors—characterized by a pungent, sour, and putrid smell—are the primary indicator of process failure and directly impact product quality and environmental compliance. To ensure consistency and minimize subjectivity, all samples were evaluated immediately upon collection by a panel of five trained assessors. Every three samples with the same rancid level were grouped together as shown in Table 1. Based on the conventional SWP food production system, we conducted a study on the microbiota (Figure 1B). It is important to address the problem that SWW effluent is often contaminated with rancidity, which can seriously reduce the recovery of nutrient resources and cause economic losses of feed and food. More seriously, air and water pollution around factories and downstream areas will occur. Therefore, we used a microbiological monitoring strategy to track the source of contamination (Figure 1B).

Microscopic examination of sampled SWW shows some bacteria are in the SWW influent and the bacteria are enriched in the effluent. All SWW influent samples were free of rancid odor, which is likely attributed to the direct and rapid flow of SWW from the upstream SPI production line into the buffer tank via pipelines. In contrast, the effluent samples exhibited varying degrees of rancidity. Therefore, we suspected that some special bacteria should be associated with the rancid pollution of the SWW effluent and the differences of these communities may be informative for such rancidity. The sampled SWW of eight groups (Table 1) were then subjected to bacterial community analysis and the rationality of the grouping was evaluated to be rational by ANOSIM analysis (Figure S1). After quality filtering and trimming, a total of 1,030,346 sequences from 24 samples were obtained from 16S rRNA gene amplicon sequencing, with an average length of 421 bp. To obtain the clearest functional gene differences under extreme phenotypes, effluent samples from severely rancid SWW (CE group) and normal SWW (UE group) were subjected to metagenomic sequencing. This yielded an average library size of 13.23 Gbp (ranging from 12.06 to 14.4 Gbp), with 98.0% (range: 97.8%–98.3%) of the bases falling within the assembled fraction (Table S1).

**FIGURE 2:**
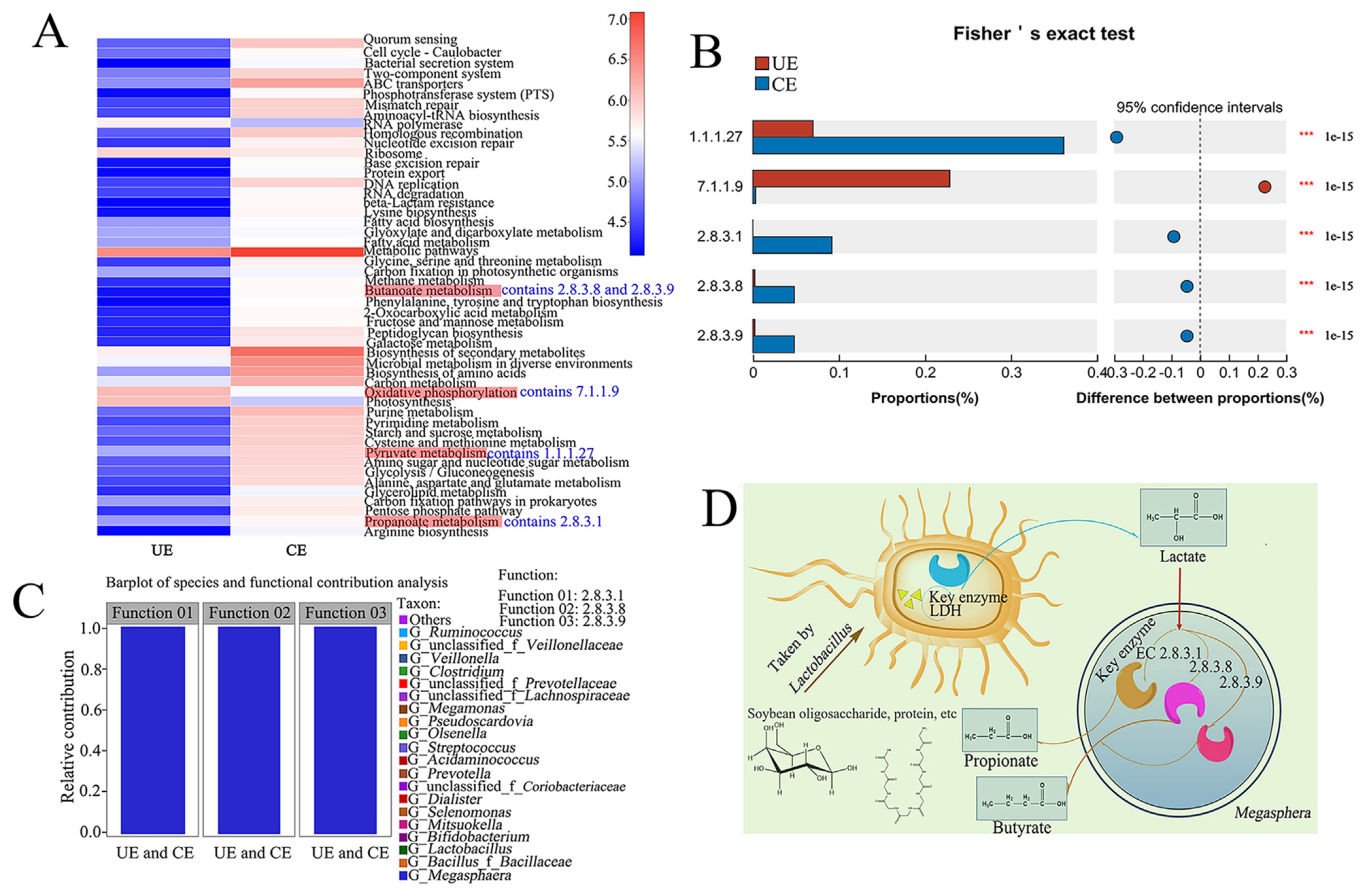
Metagenomic analysis of SWW effluent groups. (A) Heatmap of metabolism pathway level in UE and CE (butanoate metabolism, oxidative phosphorylation, pyruvate metabolism, and propanoate metabolism are marked by red); (B) difference test of related key genes at KEGG enzyme level; (C) barplot of species and functional contribution analysis about genes encoding key enzymes involved in the synthesis of propionate and butyrate in UE and CE; (D) proposed pathway for propionate and butyrate synthesis in the CE community. UE represents the SWW effluent group without rancidity and CE for the effluent group with severe rancidity. Each group contained three biological replicate samples.

We then compared the metabolic pathway genes of top 50 abundance between two metagenome sequenced groups (Figure 2A). Apparently, compared with the CE samples, there were some metabolic pathways with higher gene abundance in UE samples. Gene abundance of the oxidative phosphorylation metabolism with reads number 1.38 × 10^6^ detected in UE was much higher than that in CE (3.59 × 10^5^). Consistently, in oxidative phosphorylation metabolism, the gene abundance of key enzyme cytochrome oxidase (EC7.1.1.9) responsible for reducing oxygen to H_2_O was higher in UE (0.23%) than in CE (0.0031%). In combination with the decreased DO values detected in the rancid effluents (Table 1), we speculated that an anaerobic environment might exist in the rancid polluted SWW effluents.

### 3.2 The Microbe and Its Metabolites Responsible for the Rancidity SWW Effluent

Since the pollution produced an acidic and putrid odor, we further focused on those genes that contribute to the organic acid metabolism. Overall, the CE group gained a higher read number of pyruvate metabolism genes (8.37 × 10^5^) than UE (reads number 1.24 × 10^5^). In pyruvate metabolism genes, the gene abundance of key lactate dehydrogenase (EC1.1.1.27, LDH) decisive for the lactate synthesis showed much higher proportion (0.36%) in UE than that of CE community (0.079%) (Figure 2B), indicating lactic acid consumption occurred in rancid SWW effluents. Moreover, the gene numbers for both propionate metabolism (reads number value 4.46 × 10^5^ in CE versus 1.04 × 10^5^ in UE) and butyrate metabolism (reads number value 4.2 × 10^5^ in CE versus 1.56 × 10^4^ in UE) were higher in CE than UE. Concretely, the relative gene abundance of EC2.8.3.1 (propionate‐CoA: lactoyl‐CoA transferase) was higher in the CE group, with 0.092% proportion while not detected in the UE group. In the pathway of propionate synthesis, EC2.8.3.1 catalyzes lactate to lactoyl‐CoA and launches the propionate synthesis pathway from lactate. Also, it is responsible for transforming propionate‐CoA to propionate, the last and key step of propionate synthesis (Prabhu et al. 2012). Meanwhile, similar difference for genes encoding key enzymes for butyrate synthesis, butyryl coenzyme A transferase (EC2.8.3.8) and butyryl‐CoA‐acetoacetate CoA‐transferase (EC2.8.3.9) (Miller and Jenesel 1979; Diez‐Gonzalez et al. 1999; Duncan et al. 2002), were observed between CE and UE groups, with relative abundance of 0.048% in CE while only 0.002% detected in the UE community (Figure 2B).

To validate these gene differences at the macroscopic level, HPLC was used to monitor the content of organic acids in each group of SWW (Figure 4G). The average content of lactate in the UE samples was 8.73 ± 0.97 g L^−1^, while only a hint of butyrate (0.1 ± 0.01 g L^−1^) and propionate (0.33 ± 0.02 g L^−1^) were detected. It is worth noting that the lactate concentrations of these effluents showed a regular decline (6.68 ± 0.55 for SE, 5.31 ± 0.41 for ME, 2.65 ± 0.24 g L^−1^ for CE) with the increase of effluent pollution. In contrast, with the increase of the rancidity pollution level, the concentrations of propionate ranged from 1.15 ± 0.09 to 3.59 ± 0.28 g L^−1^ in three rancid polluted effluent groups, which is 3.5–10.9 folds higher than that of the normal SWW effluent (UE, 0.33 g L^−1^). Similarly, compared with the normal SWW effluent (UE group), more than 12‐fold butyrate was detected in those rancid SWW effluent samples, and displayed a gradual accumulation with the increased level of rancid pollution (from 1.24 ± 0.13 to 4.25 ± 0.2 g L^−1^). Therefore, all the evidence confirmed that the rancid substances in the polluted SWW effluents are propionate and butyrate, which were derived from the lactate metabolism.

To identify the microbial culprit responsible for propionate and butyrate production, a species barplot and functional contribution analysis of genes encoding key enzymes involved in propionate and butyrate synthesis were performed at the genus level. Strictly anaerobic *Megasphera* exhibited 100% contribution to the gene abundance of EC2.8.3.1, EC2.8.3.8, and EC2.8.3.9 in the CE samples, indicating the accumulations of butyrate and propionate were attributed to the metabolism of *Megasphera* (Figure 2C). This agrees well with previous findings that *Megasphera* can produce large amounts of short chain fatty acids (SCFAs) such as butyrate and propionate through anaerobic fermentation (Huang et al. 2016; Shahab et al. 2020), with stinky and unpleasant smell (O'Neill and Phillips 1992; Yan et al. 2014; Simeoli et al. 2017). Therefore, we can conclude that the presence of bacteria *Megasphera* in SWW effluents resulted in contamination, which in turn hindered subsequent resource recovery.

### 3.3 Bacterial Community Dynamics in Rancid SWW Effluents

**FIGURE 3:**
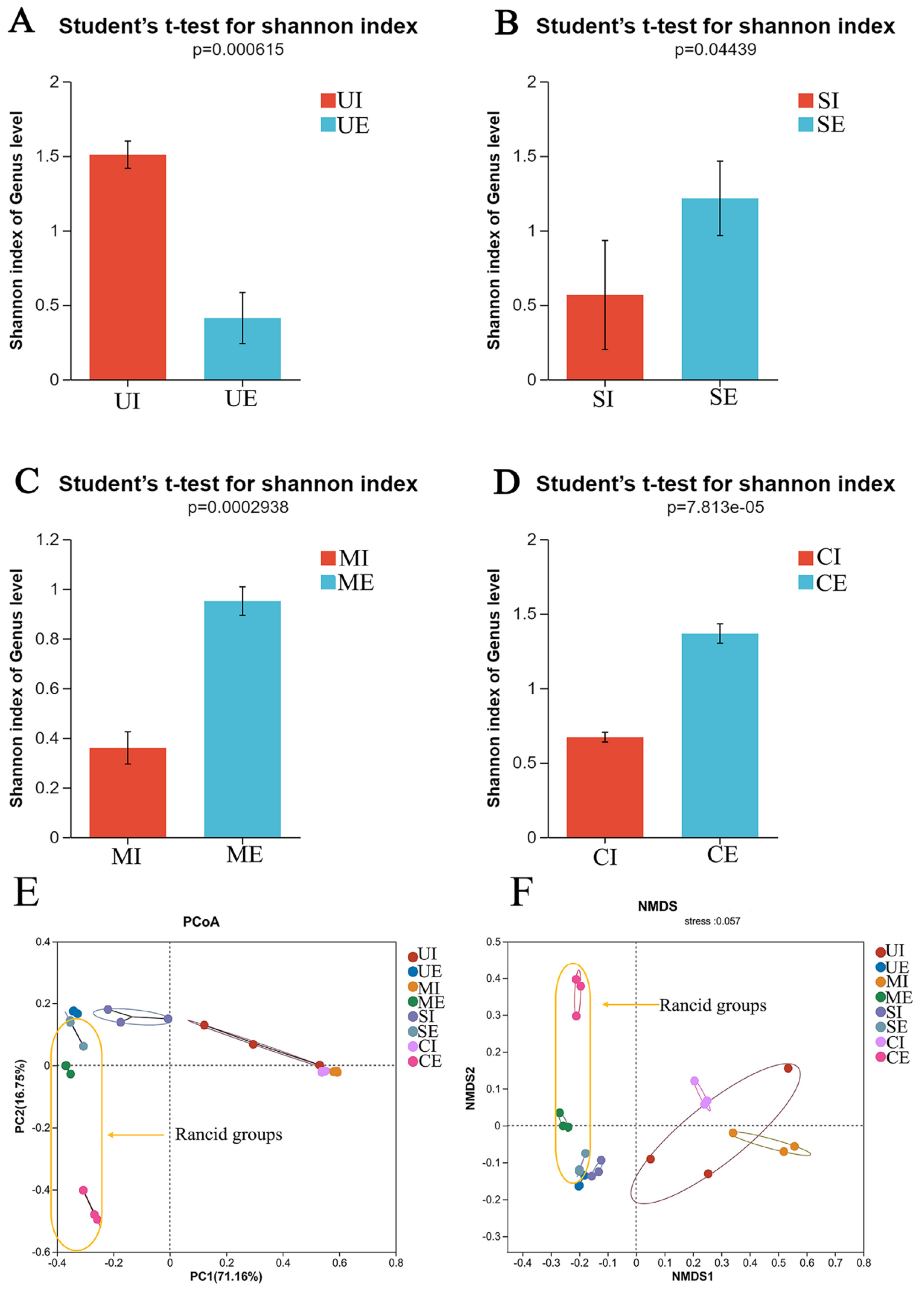
Alpha diversity analysis of SWW groups with different rancid levels (A–D), PCoA (E), and NMDS (F) analysis of the microbial community at genus level. The rancid groups are marked by orange circles and arrows. UI, SI, MI, and CI are the influent groups, which correspond to the effluent groups UE, SE, ME, and CE, respectively. UE represents the effluent group of SWW without rancidity, SE for the effluent group with slight rancidity, ME for the effluent group with moderate rancidity, and CE for the effluent group with severe rancidity. Each group contained three biological replicate samples. All the influent samples have no rancid odor, but the effluent samples have different degrees of rancid odor.

In order to summarize the variation rules of bacterial flora in SWW and provide ideas for pollution control, the bacterial flora in different groups of SWW were monitored. The mean values of every Alpha diversity index in each group were recorded in Table S2. The good's coverage values indicated the sequencing depth was sufficient to represent the diversity level of bacteria. Shannon index was calculated to compare the species diversity of each group at the genus level (Allen et al. 2009), and a remarkable difference of bacterial diversity was observed between the normal and rancid effluent samples (Figure 3A–D). In the normal SWW effluent (UE), the species diversity is significantly lower than the corresponding influent sample (UI). In contrast, the bacterial diversities of all rancid effluents are much higher than their influent samples (*p* < 0.05). This implies that more complex communities were developed in these rancid SWW effluents, causing a microbial contamination in SWW effluent and promoting the occurrence of rancidity. PCoA and NMDS analyses were further used to analyze the bacterial community differences among groups at the genus level. The result suggested a remarkable community difference between the rancid SWW groups (SE, ME, CE) and the normal SWW groups (UI, UE, SI, MI, CI), perhaps due to the emergence of new genera in the rancid SWW group (Figure 3E–F).

**FIGURE 4:**
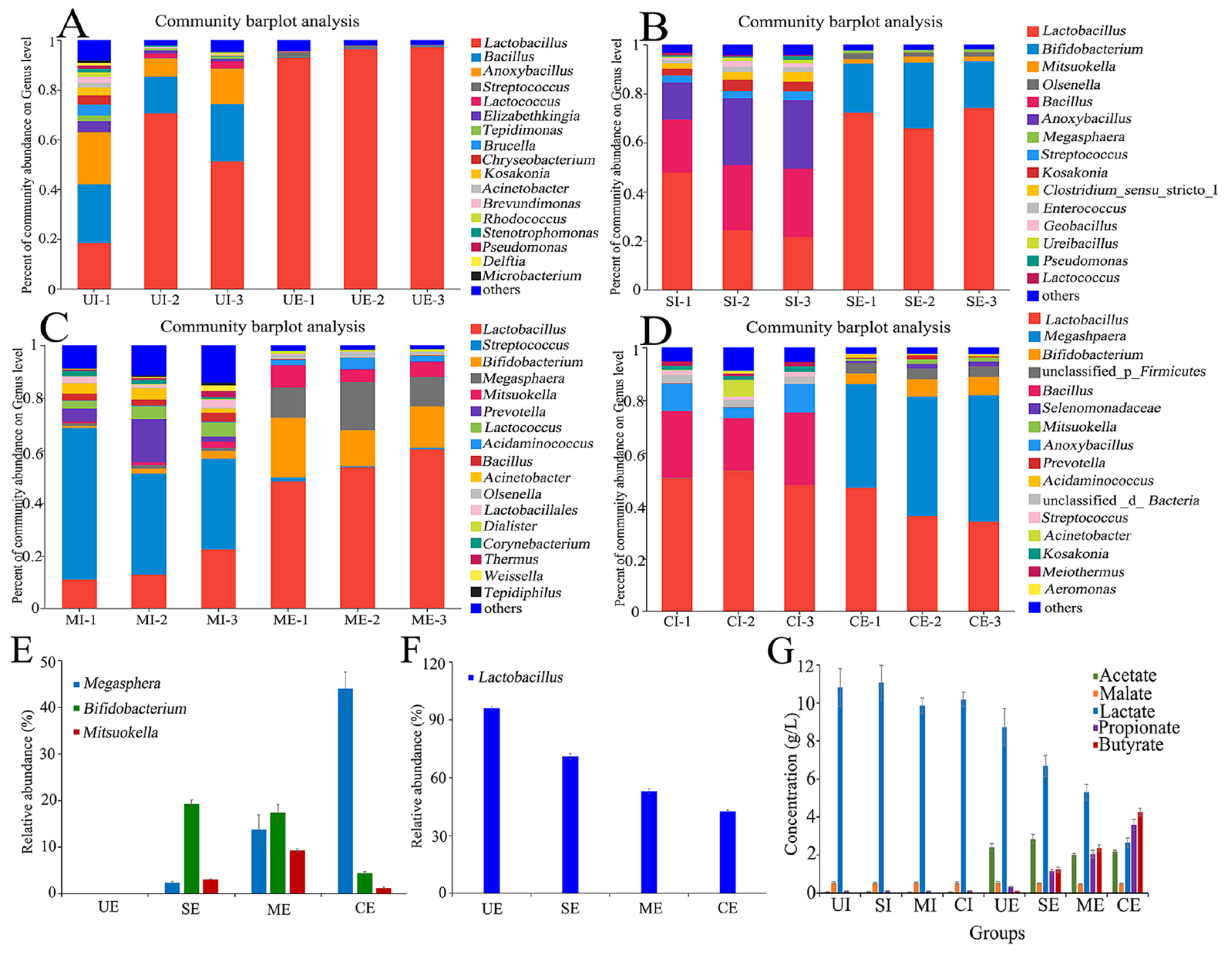
Bacterium abundance at genus level and organic acid content of SWW influent and effluent groups. Bacterium abundance in UI and UE (A), SI and SE (B), MI and ME (C), CI and CE (D) at the genus level. The abundance of three obligate anaerobes (E) and *Lactobacillus* (F) in SWW effluent samples at different rancidity levels. The content of organic acids in SWW influent and effluent samples (G). UE represents SWW effluent group without rancidity, SE for effluent group with slight rancidity, ME for effluent group with moderate rancidity, and CE for the effluent group with severe rancidity. Each group contained three biological replicate samples. −1, −2, and −3 represent the three samples in the same group.

To gain more insight into the bacterial diversities of SWW, the community abundances of these groups were compared at genus level. In normal SWW, *Lactobacillus* strains were dominant with 94.7% abundance in the effluent community of UE samples, which was much higher than that in UI (39.0%) (Figure 4A). Differently, in the effluent samples with increased level of rancid pollution, the predominance of *Lactobacillus* gradually decreased from 94.7% to 42.9% abundance (Figure 4F). The decline of *Lactobacillus* agreed well with the decreased concentration of lactate in rancid effluents (Figure 4G). In contrast to the decreased abundance of *Lactobacillus*, the obligate anaerobic *Mitsuokella*, *Bifidobacterium*, especially *Megasphera*, showed a striking abundance in rancid effluents (Figure 4E). In SE effluent samples, the relative abundance of *Megasphera* accounted for 3.4% of all bacteria (Figure 4B), and then became much more abundant in higher rancidity samples of ME (Figure 4C, 13.6%) and then CE samples (Figure 4D, 44.0%). *Bifidobacterium* and *Mitsuokella* were absent in the UE samples but appeared in all rancid effluents. However, the abundance of *Bifidobacterium* (UE: 0%; SE: 19.3%; ME: 17.4%; CE: 4.3%) exhibited an opposite trend to that of *Megasphera*, while the abundance of *Mitsuokella* (UE: 0%; SE: 3.1%; ME: 9.26%; CE: 1.2%) showed no regularity with increasing rancidity (Figure 4E). This indicates that *Megasphera* can be identified as the sole anaerobic bacterium responsible for the rancidity of SWW effluent. In SWW influents and normal effluents, there are almost no *Megasphera*, *Bifidobacterium*, and *Mitsuokella*. In combination with the decreased DO values of rancid effluents (Table 1) and main bacterial flora variation (Figure 4B–E), it can be inferred that a similar lactate‐driven DF (dark fermentation) system was developed in rancid polluted effluents (Lorenz and Gentile 2014; Chen et al. 2019; Lacroux et al. 2023). Anaerobic environment and lactate created favorable conditions for the growth of *Megasphera* species (Kung Jr and Hession 1995; Mackie et al. 2002), and consequently resulted in the outbreak of rancid pollution (Figure 2D and Figure S3). Therefore, inhibiting the growth of the genus *Megasphera* is crucial for preventing and controlling rancidity pollution in SWW effluents.

### 3.4 Proposing a Biological Approach to Suppress *Megasphera* in Rancid SWW Effluent

Based on the results of microbe and gene monitoring of SWW, it is evident that *Megasphera* is the culprit for causing the rancid pollution of SWW effluent by producing acidic pollutants of propionate and butyrate (Figure S3). Therefore, the most economical and environmentally friendly approach to eliminate rancid pollution is to apply microbial interactions to inhibit the growth of *Megasphera*. Coincidentally, the bottom of the buffer tank harbors much deposited bioactive sludge (Figure 1), which serves as an ideal colonization site for probiotics. Thus, only a one‐time inoculation of a sufficient quantity of *Megasphera*‐inhibiting probiotic with surface colonization capability is required to establish its presence.

Regarding the selection of probiotics capable of colonization and inhibiting *Megasphera*, we conducted a literature review. According to a previous study, after feeding suckling pigs with *E. faecium* , the abundance of *Megasphera* in the intestine of suckling pigs was significantly decreased (Li et al. 2017). As a probiotic, *E. faecium* has the ability to colonize the intestinal surface and is widely used as a feed additive in animal production; it can produce substances such as bacteriocins and hydrogen peroxide (Moy et al. 2004; El‐Ghaish et al. 2015), which may also inhibit strict anaerobes like *Megasphera*. These evidences inspired us to introduce laboratory‐stored *E*. *faecium* LBSW (identified as *E. faecium* SF68 through 16S rDNA sequence alignment) into the rancid polluted bacterial flora.

It is noteworthy that while *E. faecium* serves as a probiotic, it is also an opportunistic pathogen, necessitating careful consideration for use in feed and food industries. However, *E. faecium* SF68 has been proven to be a safe and effective probiotic preparation (Greuter et al. 2020). It lacks virulence factors, and studies have typically failed to detect major enterococcal virulence genes (such as *cyl*, *gelE*, and *esp*) in *E. faecium* SF68 (Eaton and Gasson 2001). It functions via colonization rather than invasion and is described as a “commensal” strain capable of colonizing the intestinal surface and coexisting peacefully with the host without generally causing systemic infections by invading the bloodstream or deep tissues (Ghazisaeedi et al. 2022). Therefore, selecting *E*. *faecium* LBSW for application in the feed industry is feasible.

Considering Shandong Lvbang Bio‐tech company's SWP production line includes subsequent drying and sterilization steps (Figure 1) at temperatures exceeding 120°C for approximately half an hour, any residual *E*. *faecium* LBSW in the resulting protein residue would likely be inactivated. Consequently, the produced SWP holds potential for application in the food industry. Nevertheless, for complete compliance and application in the food sector, future research should further conduct live bacteria detection and compositional analysis of the product. Additionally, deeper research and development or screening for new, completely non‐toxic probiotics capable of inhibiting the *Megasphera* genus are recommended in future study.

### 3.5 Effectiveness of the Proposed Biological Interaction Approach

**FIGURE 5:**
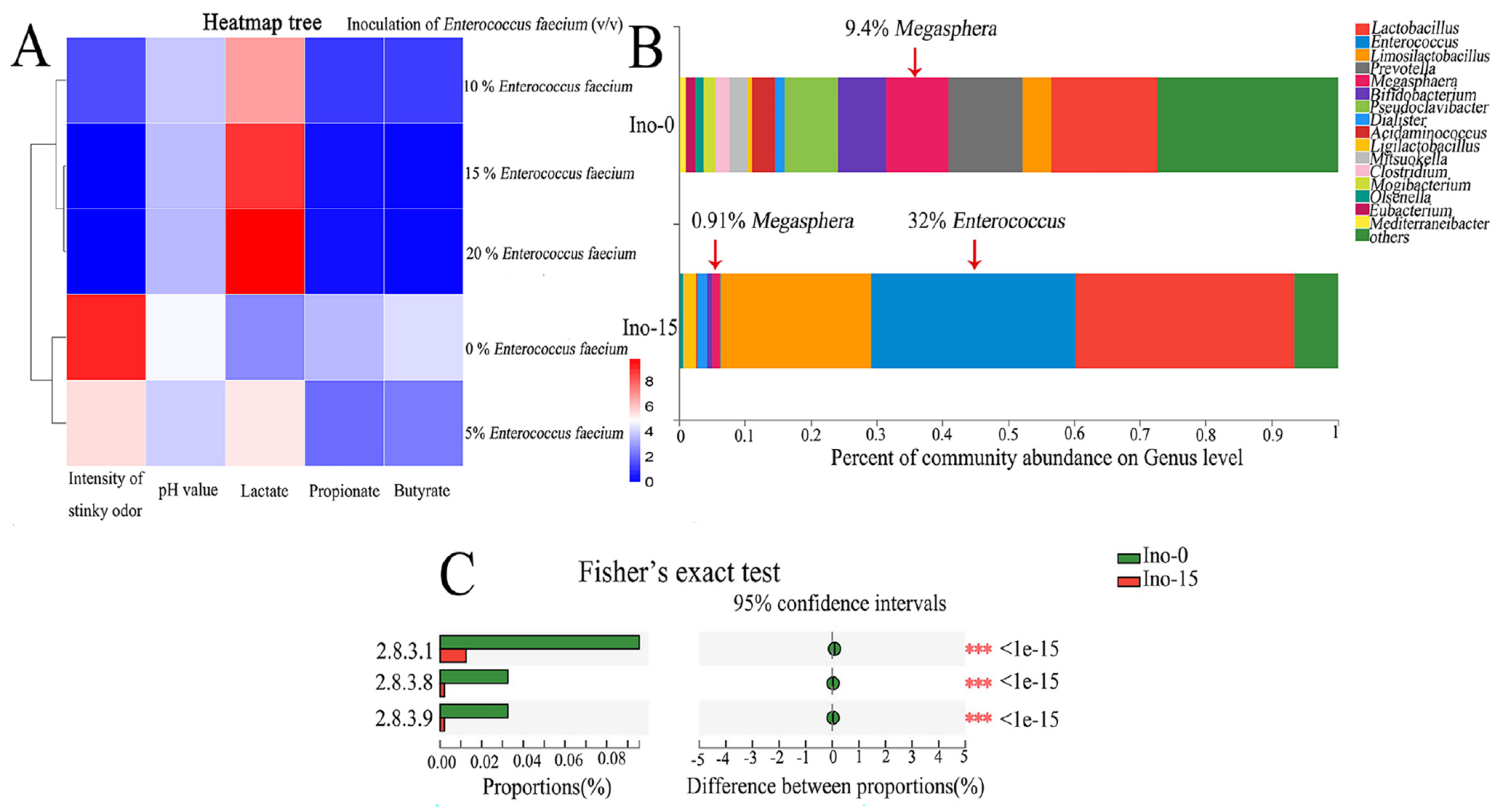
Metagenomic analysis of cocultures *E. faecium* LBSW and *Megasphera* sp. LY. (A) Parameters of 3‐day cocultures, with initial inoculation of *E*. *faecium* LBSW in different content and 2% (v/v) *Megasphera* sp. LY using SWW influent as medium; (B) Community barplot analysis at genus level; (C) Fisher's exact test analysis on genes encoding key enzymes involved in the synthesis of propionate and butyrate between the groups Ino‐0 and Ino‐15. Ino‐0 means the 3‐day cocultures, with initial inoculation of 0% (v/v) *E. faecium* LBSW and 2% *Megasphera* sp. LY using SWW influent as medium; Ino‐15 means the 3‐day cocultures, with initial inoculation of 15% (v/v) *E. faecium* LBSW and 2% *Megasphera* sp. LY using SWW influent as medium. Groups Ino‐0 and Ino‐15 each contained three biological replicate samples.

To simulate rancid pollution, we isolated the *Megasphera* sp. LY (100% sequence identity with 16S rDNA of *Megasphera elsdenii*) from the rancid effluent and inoculated it into the SWW influent. With an inoculation of 2% (v/v) of *Megasphera* sp. LY and 0% *E. faecium* LBSW, the SWW influents became seriously rancid after 3 days of co‐cultivation (marked as Ino‐0). Expectedly, after co‐cultivation with 5%–20% *E. faecium* LBSW, the growth of *Megasphera* strain was significantly inhibited and rancid odor was remarkably alleviated (Figure 5A,B). Particularly, complete inhibition was observed after the supplementation of 15% or more *E. faecium* LBSW after 3 days of co‐cultivation (mark the coculture as Ino‐15). Although a 15% inoculation ratio of *E. faecium* LBSW is relatively high, in practical industrial applications, a single inoculation is sufficient to establish colonization in the activated sludge at the bottom of the buffer tank, eliminating the need for subsequent supplementation.

Meanwhile, propionate and butyrate contents in these samples were analyzed. Consistently, 1.8 g L^−1^ propionate and 1.1 g L^−1^ butyrate were detected in the rancid polluted Ino‐0, while no corresponding HPLC peak of propionate or butyrate was observed in normal Ino‐15 (Figure 5A and Figure S2). Given that *E. faecium* LBSW is facultatively anaerobic and was introduced at a higher inoculum ratio, it rapidly depleted the dissolved oxygen, creating an anaerobic niche. The subsequent lack of *Megasphaera* proliferation—even in the absence of oxygen—confirms the inhibitory effect exerted by *E. faecium* LBSW.

To further elucidate such inhibition and the anabolism of two pollutants propionate and butyrate, metagenomic sequencing was conducted for the groups Ino‐0 and Ino‐15 and yielded an average library size of 12.2 Gbp (range, 11.5–12.9 Gbp), with 99.3% of the bases (range, 99.2%–99.3%) in the assembled fraction (Table S3). Expectedly, key genes for the synthesis of propionate and butyrate, namely EC2.8.3.1 (0.096% in Ino‐0 versus 0.008% in Ino‐15), EC2.8.3.8 (0.033% in Ino‐0 versus 0.002% in Ino‐15), and EC2.8.3.9 (0.032% in Ino‐0 versus 0.002% in Ino‐15) all significantly decreased in Ino‐15 (Figure 5C).

The community structure of Ino‐0 is complex, with main members of *Lactobacillus* (16.0%), *Prevotella* (11.3%), *Megasphera* (9.4%), *Pseudoclavibacter* (8.14%), *Bifidobacterium* (7.3%), *Limosilactobacillus* (4.3%), and *Mitsuokella* (2.68%). As expected, the structure of the bacterial community in Ino‐15 was greatly altered and simplified, with three facultative anaerobes, *Lactobacillus* (33.2%), *Enterococcus* (32%), and *Limosilactobacillus* (22.8%), dominating. On the contrary, only 0.94% of *Megasphera* and 0.7% of *Bifidobacterium* were detected, and the obligate anaerobes *Petrella* and *Mitsuokella* disappeared (Figure 5B). These differences agreed well with the inhibited rancid pollution of Ino‐15 and provided solid support for the inhibited growth of *Megasphera* by *E. faecium* LBSW. Worth noting, when comparing two normal communities UE and Ino‐15 without rancidity (Figures 4A and 5B), we found an interesting consistency of lactic acid bacteria dominance in both bacterial communities. In the UE group, around 90% *Lactobacillus* was dominant in the community and produced high contents of lactate. In the reconstructed community of Ino‐15, three dominating genera of *Lactobacillus*, *Enterococcus*, and *Limosilactobacillus* were all lactic acid bacteria. Therefore, as an environmentally friendly strategy, the inoculation of *E. faecium* LBSW can lead the content of propionate and butyrate to decline, and consequently eliminate the rancid pollution of SWW effluent (Figure 1B).

### 3.6 Practical Application Strategies for Mitigating Rancidity in SWP Production Factories

Considering the cost and energy consumption in practical applications, this study proposes three strategies:

(1) Installing high‐throughput aeration equipment throughout the buffer tank (2400 m^3^) to create an aerobic environment that disrupts the growth of *Megasphera*. However, this approach entails high energy consumption and equipment costs.

(2) Installing local aeration equipment in the middle section of the buffer tank. Although this reduces equipment costs, the presence of aeration dead zones limits the effectiveness of rancidity inhibition. Moreover, continuous operation results in non‐negligible energy consumption.

(3) Employing microbial intervention using probiotic *E*. *faecium*. LBSW. This strategy involves halting production for two days to inoculate a large volume of *E. faecium* LBSW, allowing it to colonize the activated sludge within the buffer tank once and for all. Afterward, no further additions are required. Local aeration is only re‐activated as a supplementary measure when rancidity is severe and microbial intervention alone is insufficient to control *Megasphera*.

To address this obstacle economically and energy‐efficiently, this study adopts Strategy (3). Although the biosafety of *E. faecium* LBSW in food systems requires careful assessment, the SWP recovered in this study is primarily intended for the feed industry. Furthermore, the subsequent high‐temperature drying and sterilization processes confer potential for application in the food industry as well.

Implementation of Strategy (3) is as follows: A large‐scale, single‐dose inoculation of *E. faecium* LBSW is administered into the activated sludge at the bottom of the SWW. To verify successful colonization, PCR was used to amplify the 16S rDNA of *E. faecium* LBSW from the sludge samples. The large size of *Megasphera* strain provides strong operability for monitoring the occurrence of rancidity through the microscopic examination every day. Therefore, when large *Megasphera* cells appear in the microscopic field of view but are not numerous, the factory can activate the local aeration system to increase the DO level in the SWW. This can inhibit the growth of the strictly anaerobe *Megasphera* while promoting the proliferation of *E. faecium* LBSW (as the latter is a facultative anaerobe that grows better under aerobic conditions). The local aeration device can be turned off to save energy when no large *Megasphera* is observed in the microscopic field of view. This strategy would have a synergistic effect in controlling the growth of *Megasphera* by altering the anaerobic conditions to inhibit *Megasphera* and simultaneously creating a better environment for *E. faecium* LBSW (Figure 1B). Expectedly, the application of such a combined strategy of microbial interaction and aeration was effective on an industrial scale. After one year of operation, this combined strategy not only eliminated the rancid pollution of SWW completely but also gained a stable and higher recovery rate of SWP feed with more protein content (8500 tons nutrients per year with 50% crude protein).

## 4 Conclusions

In this study, we elucidated the origins of rancidity SWW effluent and further constructed a strategy for the efficient production of SWP feed from SWW. Through microbial monitoring and metagenomic analysis, we explained why SWW effluent is susceptible to rancid pollution. In addition, this study solved the problem that SWW effluent is easy to be polluted by *Megasphera* and its metabolites, which has a certain positive effect on air quality, water quality and ecological environment energy flow around feed and food factories. The relationship between microbial community metabolism and the formation of environmental pollutants proposed in this study (Figure S3) can also shed some insights into water remediation and the development of economical control measures in other feed and food production processes. To eliminate microbial contamination and improve nutrient recovery, all that is required is the addition of biocontrol strains once and for all, and the activation of the local aeration device when needed, without the use of chemical reagents, so there is no reagent contamination. The produced protein residue product, enriched with SWP, is also expected to be applied in the food industry after subsequent high‐temperature drying and sterilization processes.

## Author Contributions

**Zhiyong Zhang:** resources, conceptualization, funding acquisition. **Chunwen Yang:** formal analysis, methodology. **Tong Mu:** methodology, visualization. **Wei Wang:** conceptualization, methodology, investigation, data curation. **Chongzhou Li:** conceptualization. **Yishu Peng:** methodology, investigation, data curation. **Jinlu Wang:** methodology, investigation. **Junhui Zhao:** formal analysis, investigation. **Yuanxiang Liu:** conceptualization, validation, formal analysis, investigation, writing – original draft. **Luhua Feng:** investigation, conceptualization, software. **Chunfang Li:** methodology, data curation. **Chunyu Yang:** resources, data curation, writing‐review and editing, supervision, project administration, funding acquisition.

## Funding

National Natural Science Foundation of China, 32570120, 42077214 and Science and Technology Projects of Yangzhou City Z2023043.

## Conflicts of Interest

The authors declare no conflicts of interest.

## Supporting information


**Figure S1:** ANOSIM analysis between groups. Compared with the between group, all other groups showed significant differences (*p* < 0.05). The X‐axis is the distance value within or between groups, the box corresponding to between represents the distance value of the difference between the groups, and the remaining boxes represent the difference distance value within the group; The Y‐axis scale represents the magnitude of the distance value. UI, SI, MI, and CI are the influent samples, which correspond to the effluent samples UE, SE, ME, and CE, respectively. UE represents for effluent samples of SWW without rancidity, SE for effluent samples of slightly rancid SWW, ME for effluent samples of moderately rancid SWW and CE for effluent samples of severe rancid SWW. All the influent samples have no rancid odor, but the effluent samples have different degrees of rancid odor.
**Figure S2:** HPLC analysis of organic acids of the sample Ino‐0 and Ino‐15, related to Figure 5A. A: HPLC analysis of organic acids of the sample Ino‐0; B: HPLC analysis of organic acids of the sample Ino‐15; Ino‐0 means the coculture comprised of *Enterococcus* *faecium* LBSW in 0% (v/v) inoculation content and 2% *Megasphera* sp. LY using SWW influent as medium; Ino‐15 means the coculture comprised of E. *faecium* LBSW in 15% (v/v) inoculation content and 2% *Megasphera* sp. LY using SWW influent as medium.
**Figure S3:** Schematic illustration of the metabolic networks of *Lactobacillus* and *Megasphera* for the rancid pollution of SWW effluent. The influent of soybean whey wastewater (SWW) flows through a buffer tank. When the condition in the tank is extremely anaerobic, *Megasphera* will interact with *Lactobacillu* *s*, producing smelly short‐chain fatty acids thus resulting in rancidity of effluent of soybean whey wastewater.
**Table S1:** Alpha diversity index of bacterial community.
**Table S2:** Characteristics of metagenomic libraries of UE and CE groups.
**Table S3:** Characteristics of metagenomic libraries of Ino‐0 and Ino‐15 groups.

## Data Availability

The data that support the findings of this study are available on request from the corresponding author. The data are not publicly available due to privacy or ethical restrictions.
