# Supplementary material for: Sources of Microbial and Organic Contaminants in the Production of Soybean Whey Protein for Feed and Potential Food Applications
Source: Food Sci Nutr. 2026 Apr 29;14(5):e71709. doi: 10.1002/fsn3.71709 (PMC13126613; doi:10.1002/fsn3.71709)
Supplement: Supplementary file 1 — Figure S1: ANOSIM analysis between groups. Compared with the between group, all other groups showed significant differences (p < 0.05). The X‐axis is the distance value within or between groups, the box corresponding to between represents the distance value of the difference between the groups, and the remaining boxes represent the difference distance value within the group; The Y‐axis scale represents the magnitude of the distance value. UI, SI, MI, and CI are the influent samples, which correspond to the effluent samples UE, SE, ME, and CE, respectively. UE represents for effluent samples of SWW without rancidity, SE for effluent samples of slightly rancid SWW, ME for effluent samples of moderately rancid SWW and CE for effluent samples of severe rancid SWW. All the influent samples have no rancid odor, but the effluent samples have different degrees of rancid odor. Figure S2: HPLC analysis of organic acids of the sample Ino‐0 and Ino‐15, related to Figure 5A. A: HPLC analysis of organic acids of the sample Ino‐0; B: HPLC analysis of organic acids of the sample Ino‐15; Ino‐0 means the coculture comprised of Enterococcus faecium LBSW in 0% (v/v) inoculation content and 2% Megasphera sp. LY using SWW influent as medium; Ino‐15 means the coculture comprised of E. faecium LBSW in 15% (v/v) inoculation content and 2% Megasphera sp. LY using SWW influent as medium. Figure S3: Schematic illustration of the metabolic networks of Lactobacillus and Megasphera for the rancid pollution of SWW effluent. The influent of soybean whey wastewater (SWW) flows through a buffer tank. When the condition in the tank is extremely anaerobic, Megasphera will interact with Lactobacillu s, producing smelly short‐chain fatty acids thus resulting in rancidity of effluent of soybean whey wastewater. Table S1: Alpha diversity index of bacterial community. Table S2: Characteristics of metagenomic libraries of UE and CE groups. Table S3: Characteristics of metagenomic libraries of Ino‐0 and Ino‐1 [file FSN3-14-e71709-s001.docx]

*Supporting information for*

**Sources of Microbial and Organic Contaminants in the Production of Soybean Whey Protein for Feed and Potential Food Applications**

Yuanxiang Liu^1^, Wei Wang^1^, Yishu Peng^1^, Luhua Feng^1^, Zhiyong Zhang^2^, Junhui Zhao^2^, Chunwen Yang^2^, Tong Mu^2^, Jinlu Wang^2^, Chunfang Li^1^, Chunyu Yang^1^*

1. State Key Laboratory of Microbial Technology, Institute of Microbial Technology,

Shandong University, Qingdao, P. R. China

1. Shandong LvBang Bio-tech company, Binzhou, P. R. China

*Corresponding author: Prof. Chunyu Yang

Telephone: 86-0532-58631501

Fax: 86-0532-58631501

Email: 1334270355@qq.com


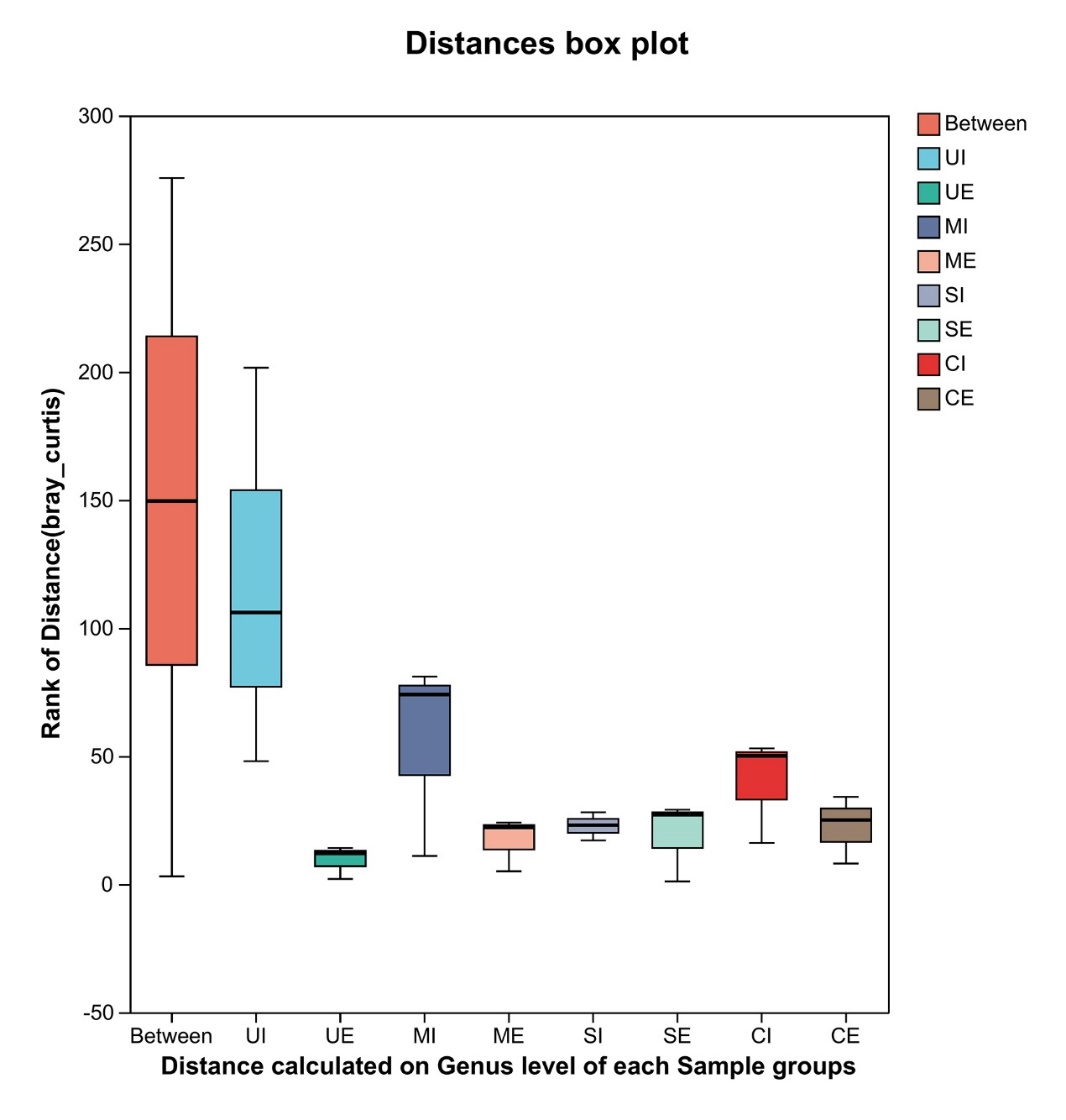
**Figure. S1.** **ANOSIM analysis between groups**. Compared with the Between group, all other groups showed significant differences (P<0.05). The X-axis is the distance value within or between groups, the box corresponding to Between represents the distance value of the difference between the groups, and the remaining boxes represent the difference distance value within the group; The Y-axis scale represents the magnitude of the distance value. UI, SI, MI, and CI are the influent samples, which correspond to the effluent samples UE, SE, ME, and CE, respectively. UE represents for effluent samples of SWW without rancidity, SE for effluent samples of slightly rancid SWW, ME for effluent samples of moderately rancid SWW and CE for effluent samples of critically rancid SWW. All the influent samples have no rancid odor, but the effluent samples have different degrees of rancid odor.

**Figure S2. HPLC analysis of organic acids of the sample Ino-0 and Ino-15, related to Figure 5A.** A: HPLC analysis of organic acids of the sample Ino-0; B: HPLC analysis of organic acids of the sample Ino-15
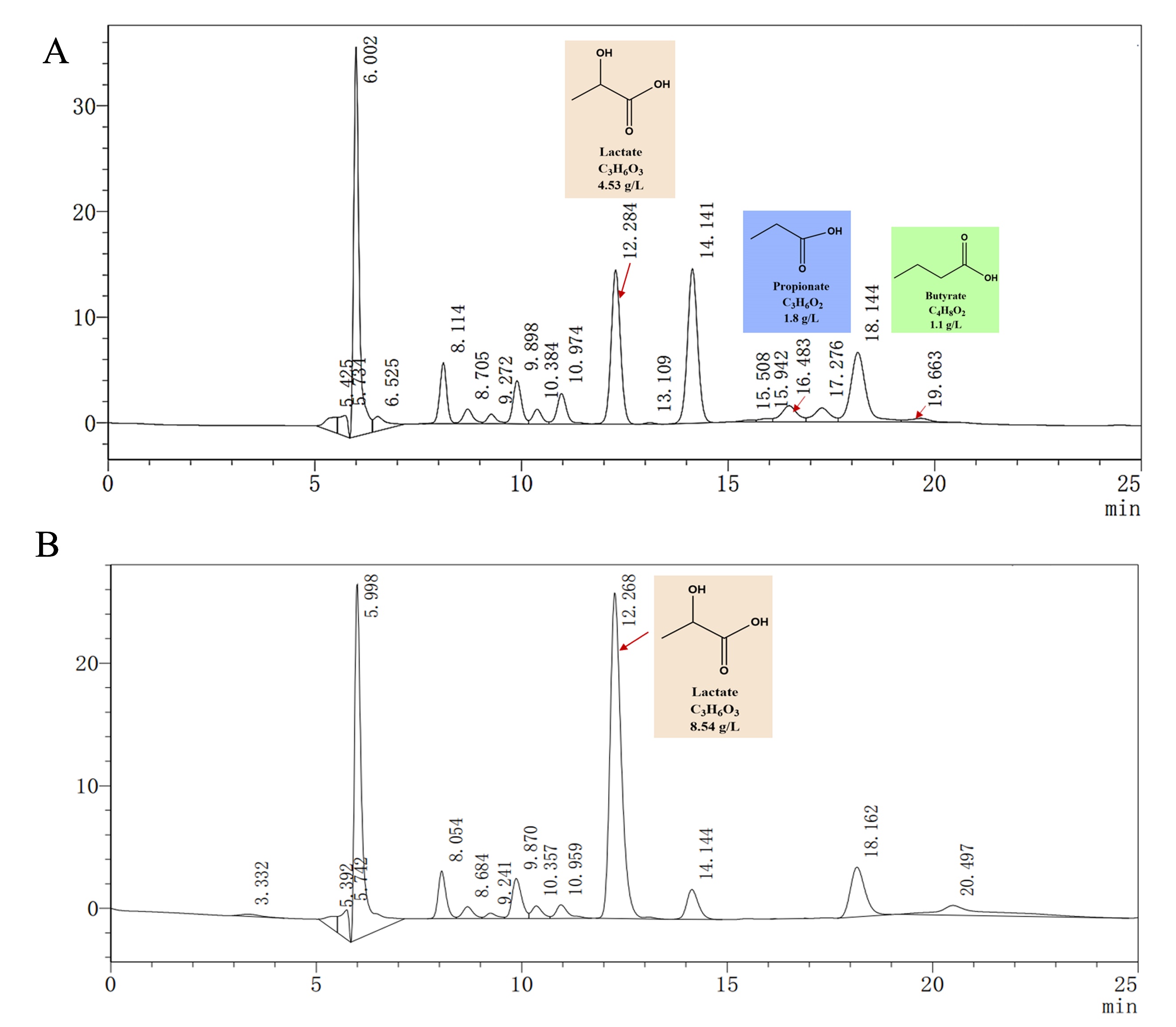
; Ino-0 means the coculture comprised of *Enterococcus* sp. LBSW in 0%(v/v) inoculation content and 2% *Megasphera* sp. LY using SWW influent as medium; Ino-15 means the coculture comprised of *Enterococcus* sp. LBSW in 15%(v/v) inoculation content and 2% *Megasphera* sp. LY using SWW influent as medium.


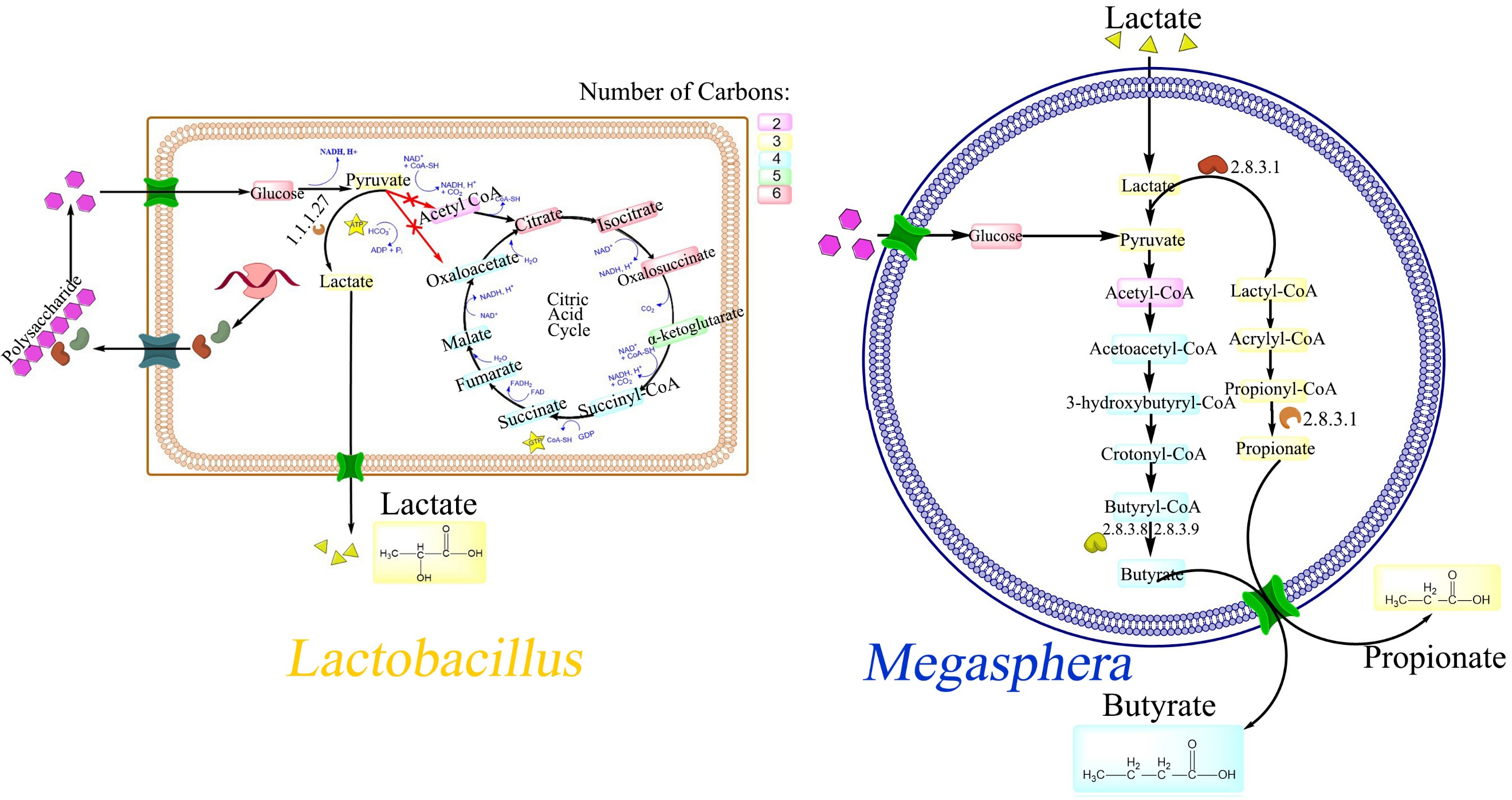


**Figure S3**. **Schematic illustration of the metabolic networks of *Lactobacillus* and *Megasphera* for the rancid pollution of SWW effluent**. The influent of soybean whey wastewater (SWW) flows through a buffer tank. When the condition in the tank is extremely anaerobic, *Megasphera* will interact with *Lactobacillus*, producing smelly short-chain fatty acids thus resulting in rancidity of effluent of soybean whey wastewater.

**Table S2** Characteristics of metagenomic libraries of UE and CE groups

| Group | Sample size  (Gbp) | Assem. Fraction  (%) | Assy. Size  (Mbp) | Contigs  >50 kbp | Contigs  >100 kbp | Longest contig (kbp) | Prokaryotic gene percentage | Eukaryotic gene percentage |
| --- | --- | --- | --- | --- | --- | --- | --- | --- |
| UE | 12.06 | 98.26 | 106.1 | 0 | 0 | 81.2 | 96.1% | 3.70% |
| CE | 14.4 | 97.79 | 595 | 0 | 0 | 91.7 | 98.2% | 0.96% |

**Table S1** Alpha diversity index of bacterial community

| Group | Good’s average | Shannon | Simpson | Chao1 | pd | Ace |
| --- | --- | --- | --- | --- | --- | --- |
| UI | 0.99945 | 1.508 | 0.3592 | 91.695 | 9.8955 | 89.718 |
| UE | 0.99954 | 0.412 | 0.87258 | 77.37 | 9.8239 | 78.47 |
| SI | 0.99937 | 0.56705 | 0.79933 | 127.6 | 14.553 | 146.69 |
| SE | 0.99947 | 1.2151 | 0.51717 | 153.18 | 17.726 | 146.03 |
| MI | 0.99967 | 0.35954 | 0.87829 | 46.686 | 5.2107 | 57.972 |
| ME | 0.9996 | 0.9505 | 0.54638 | 56.664 | 7.5784 | 60.184 |
| CI | 0.99953 | 0.67063 | 0.73754 | 90.621 | 1.9667 | 114.23 |
| CE | 0.99953 | 1.3787 | 0.35558 | 109.74 | 11.882 | 99.788 |

**Table S3** Characteristics of metagenomic libraries of Ino-0 and Ino-15 groups

| Group | Rancidity | Sample size  (Gbp) | Assem. fraction (%) | Assy. Size  (Mbp) | Contigs  >50 kbp | Contigs  >100 kbp | Longest contig  (kbp) |
| --- | --- | --- | --- | --- | --- | --- | --- |
| Ino-0 | rancid | 11.5 | 99.2 | 99.9 | 0 | 0 | 402 |
| Ino-15 | normal | 12.9 | 99.3 | 120.1 | 0 | 0 | 249 |
